# Skeletal stability after maxillary distraction osteogenesis or conventional Le Fort I osteotomy in patients with cleft lip and palate: A superimposition-based cephalometric analysis

**DOI:** 10.1007/s10006-024-01227-0

**Published:** 2024-02-16

**Authors:** Joakim Lundberg, Nameer Al-Taai, Eva Levring Jäghagen, Maria Ransjö, Mats Sjöström

**Affiliations:** 1 Oral and Maxillofacial Surgery, Department of Odontology, Umeå University, 90185 Umeå, Sweden; 2 Orthodontics, Department of Odontology, Umeå University, SE-90185 Umeå, Sweden and Hamdan Bin Mohammed College of Dental Medicine, MBRU University, Dubai, UAE; 3 Oral and Maxillofacial Radiology, Department of Odontology, Umeå University, 90185 Umeå, Sweden; 4 Orthodontics, Department of Odontology, Umeå University, 90185 Umeå, Sweden

**Keywords:** Distraction osteogenesis, Maxillary repositioning, Orthognathic surgery, Skeletal relapse, Cleft lip and palate

## Abstract

**Purpose:**

The aim was to assess skeletal stability after maxillary advancement using either distraction osteogenesis (DO) or conventional Le Fort I osteotomy (CO) in patients with cleft lip and palate (CLP) or cleft palate (CP) utilising a new superimposition-based cephalometric method.

**Method:**

This retrospective study included patients who were treated with DO (N = 12) or CO (N = 9). Sagittal and vertical changes after surgery, and skeletal stability at 18 months post-operatively were assessed with superimposition-based cephalometry, comparing lateral cephalograms performed pre-operatively (T0), post-operatively after CO or immediately after completed distraction in DO (T1), and at 18 months of follow-up (T2).

**Results:**

The mean sagittal movements from T0 to T2 in the DO and CO groups were 5.9 mm and 2.2 mm, respectively, with a skeletal relapse rate of 16% in the DO group and 15% in the CO group between T1 and T2. The vertical mean movement from T0 to T2 in the DO and CO groups was 2.8 mm and 2.0 mm, respectively, and the skeletal relapse rate between T1 and T2 was 36% in the DO group and 32% in the CO group.

**Conclusion:**

Sagittal advancement of the maxilla was stable, in contrast to the vertical downward movement, which showed more-extensive relapse in both groups. Despite more-extensive maxillary advancement in the DO group, the rates of skeletal relapse were similar.

## Introduction

Facial appearance, speech, feeding, teeth, jaws, and auditory perception can be affected in patients with cleft lip and palate (CLP) [1]. Different surgical procedures are used to improve the aesthetics and functional abilities, such as speech and swallowing. However, early surgical procedures, such as closure of the palate, can lead to reduction of mid-facial growth, resulting in a retrognathic and impacted maxilla [2–6]. Up to 27% of patients with CLP require maxillary repositioning with orthognathic surgery when orthodontic treatment is deemed to be insufficient [5–8].

Skeletal stability following the performance of a surgical procedure on the facial skeleton is affected by the size and direction of the surgery-associated movement. Movement of the maxilla downwards in the vertical plane entails a high risk of relapse, as compared to sagittal movement forward [9], and greater surgical movement is associated with a higher risk of skeletal relapse [10, 11]. Following maxillary advancement by Le Fort I osteotomy (CO), patients with CLP are at higher risk of skeletal relapse than patients without CLP, partly due to residual scar formation after surgical closure of the cleft and the tightness of the upper lip [11–13]. Distraction osteogenesis (DO) is a surgical technique that involves gradual extension of the facial bones, as well as the soft tissues [13–15]. Studies of the surgical movements of the maxilla in patients with cleft palate have indicated that DO can improve skeletal stability after surgery and lower the risk of skeletal relapse, as compared to patients who undergo CO [14–18].

Two-dimensional (2D) cephalometric analysis is the standard procedure for evaluating the outcomes and stability levels of orthodontic and orthognathic treatments [6, 10, 11, 14, 15, 17, 19, 20]. Unfortunately, when evaluating treatment outcomes through comparisons of cephalometric measurements performed at different time-points, the results are affected by growth-related positional changes of the reference landmarks [21]. Thus, superimposition of the follow-up cephalometric radiographs onto stable anatomical structures must be used to enable an accurate and reliable evaluation of the craniofacial growth and/or treatment-related changes over time [20–22]. However, conventional cephalometric superimposition provides only a graphical illustration of the changes over time. A new digital superimposition-based cephalometric method has recently been reported to provide valid and reliable measurements of craniofacial changes related to growth and/or treatment [20].

The aim of this retrospective study was to assess, using the new superimposition-based cephalometric method, the sagittal and vertical skeletal stability levels after maxillary advancement performed with either an intra-oral distraction device (i.e., DO) or conventional advancement in one session (i.e., CO) in patients with CLP. Our hypothesis was that DO lowers the amount of skeletal relapse compared to CO.

## Materials and methods

### Patient distribution for intervention

This retrospective study included patients who were born with CLP or cleft palate (CP) and in need of surgical correction of skeletal discrepancies. In northern Sweden, 278 patients born between 1981 and 2002 without any syndrome were diagnosed with unilateral or bilateral CLP or isolated cleft palate and treated according to the regional treatment protocol.

**Fig. 1:**
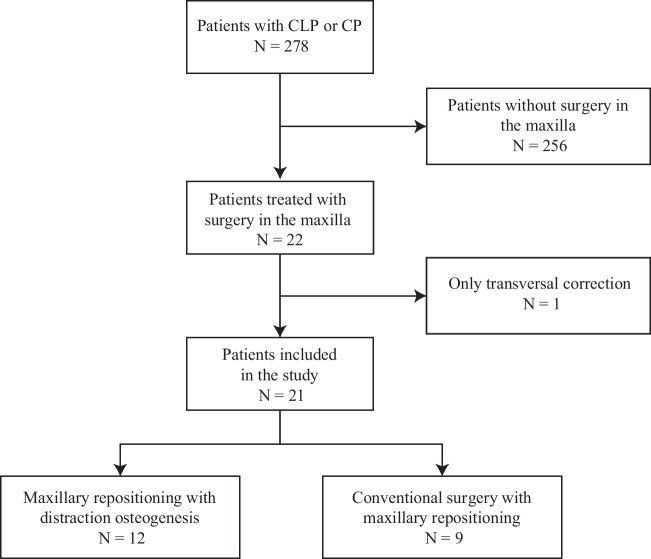
Flow chart showing the inclusion, exclusion, and withdrawal data for patients with cleft lip and palate (CLP) or cleft palate (CP)

Figure 1 presents a flow chart of the enrolment and exclusion protocols for the participants in the present study. Patients with a syndrome were excluded, as was one patient who was only treated with surgical correction of a transversal discrepancy.

Surgery was performed at the Department of Oral and Maxillofacial Surgery, Umeå University Hospital. A total of 21 (8% out of 278) of these patients received orthognathic surgery between the years 2002 and 2020; 12 patients were treated with DO and 9 were treated with CO to correct maxillary hypoplasia (skeletal discrepancy in the sagittal and/or vertical planes).

Of the 12 patients who were treated with DO, 8 (67%) were men and 4 (33%) were women, 7 (58%) had unilateral CLP, 4 (33%) had bilateral CLP, and 1 (8%) had CP. The mean age at surgery was 19.6 years (range, 14.8–23.6 years).

Of the nine patients that were treated with CO, seven (78%) were men and two (22%) were women, seven (78%) had unilateral CLP and two (22%) had CP. The mean age at surgery was 22.2 years (range, 20.0–26.2 years).

Three patients underwent surgery with a velopharyngeal flap due to velopharyngeal insufficiency prior to the orthognathic surgery (two in the DO group and one in the CO group). Bi-maxillary surgery was performed on five patients in the CO group, while all procedures in the DO group were single-jaw surgeries. Inter-positional iliac crest bone grafts were used in two of the Le Fort I osteotomies in the CO group.

A subgroup analysis was performed where the patients were divided into two age groups, the ten youngest compared to the ten oldest patients, analysing differences in skeletal relapse rate between the age groups.

### Choice of surgical technique

During the pre-operative planning, decisions as to surgical technique were based on the sagittal discrepancy between the dental arches. According to the available literature, DO is indicated for extensive maxillary advancement, whereas CO is indicated when the maxillary advancement is less-extensive [23, 24]. The DO procedure was performed on patients with a discrepancy between the dental arches that exceeded 6 mm, based on the results of Daimaruya et al. [25].

#### Distraction osteogenesis

**Fig. 2:**
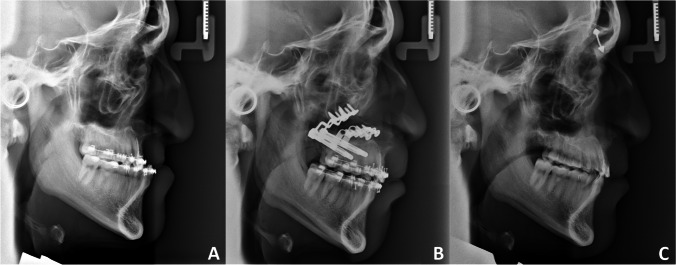
Radiographical examinations of the facial skeleton before and after treatment with DO. **A**, Lateral cephalogram prior to surgery (T0). **B**, Lateral cephalogram after surgery and advancement with the distraction device, at the start of the consolidation phase (T1). **C**, Lateral cephalogram at a mean of 18 months post-surgery (T2)

Prior to the surgery, an intra-oral distractor (DePuy Synthes) was pre-bent on a printed 3D model of the patient’s facial skeleton. The vector of movement was planned after analysing the basal discrepancy between the dental arches. Under general anaesthesia, the placement of the distractor was marked on the lateral surfaces of the maxilla. After CO, the dental part of the maxilla was mobilised before fixation to the distractor. The patient was prescribed 1.6 g phenoxymethylpenicillin to be taken three times a day for 7 days. The active distraction was initiated 7 days post-operatively (latency period). During the distraction phase, the distraction device was activated with two turns per day (one turn is equivalent to 0.5 mm of movement in the distractor), resulting in movement of 1 mm/day. The distraction phase continued until the desired occlusion was achieved, after which the skeletal sagittal relationship between the maxilla and mandible was assessed in a lateral cephalometric examination (T1). The appliance remained in place during the consolidation phase, which lasted 3–8 months post-operatively, depending on the length of the distraction and the loading situation between the dental arches. The final clinical and radiographical examinations were performed at a mean of 18 months post-distraction (T2) (Fig. 2).

#### Conventional Le Fort I osteotomy

**Fig. 3:**
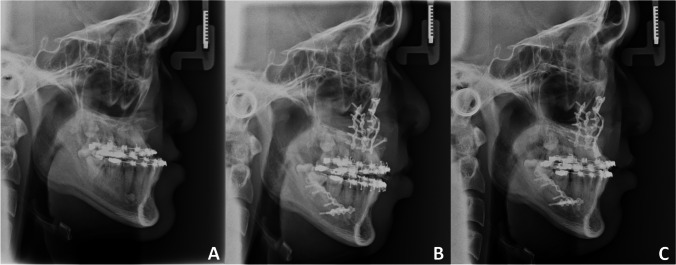
Radiographical examinations of the facial skeleton before and after treatment with CO. **A**, Lateral cephalogram prior to surgery (T0). **B**, Lateral cephalogram immediately after surgery (T1). C, Lateral cephalogram at a mean of 18 months post-surgery (T2)

Under general anaesthesia, the tooth-bearing part of the maxilla was mobilised by standard CO. The maxilla was mobilised and fixated to the mandibular dental arch using a splint in the planned position, followed by fixation to the mid-face in the planned new position with titanium mini-plates (MatrixORTHOGNATHIC™ System) The surgical outcome was assessed by lateral cephalometric examination (T1). The final clinical and radiographical examinations were performed at 18 months post-operatively (T2) (Fig. 3). For those patients in which the inter-maxillary discrepancy required surgical correction of the mandible, bilateral sagittal split osteotomy was performed.

### Cephalometric analysis

The surgical outcomes in this study were analysed using standardised cephalograms taken at T0 (pre-operatively), T1 (immediately after distraction was completed in DO and immediately after surgery in CO), and T2 (follow-up at a mean of 18 months) (Fig. 2A-C, and Fig. 3A-C). The cephalograms at T0, T1 and T2 were all acquired at a magnification of 1.1 × (in the mid-line). The linear measurements were adjusted to a standardised enlargement by 10%.

**Fig. 4:**
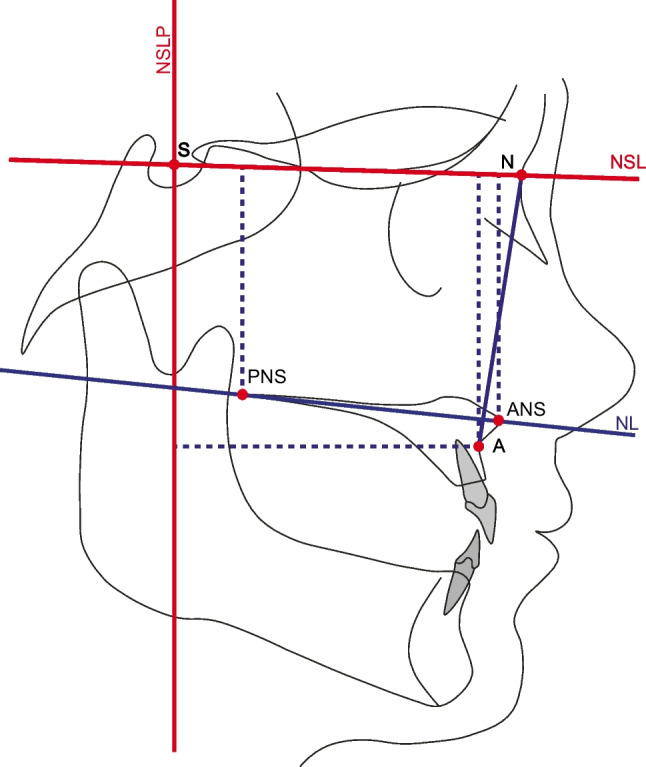
Illustration of the cephalometric landmarks, reference lines and measures used in this study. The abbreviations are explained in Table 1

**Fig. 5:**
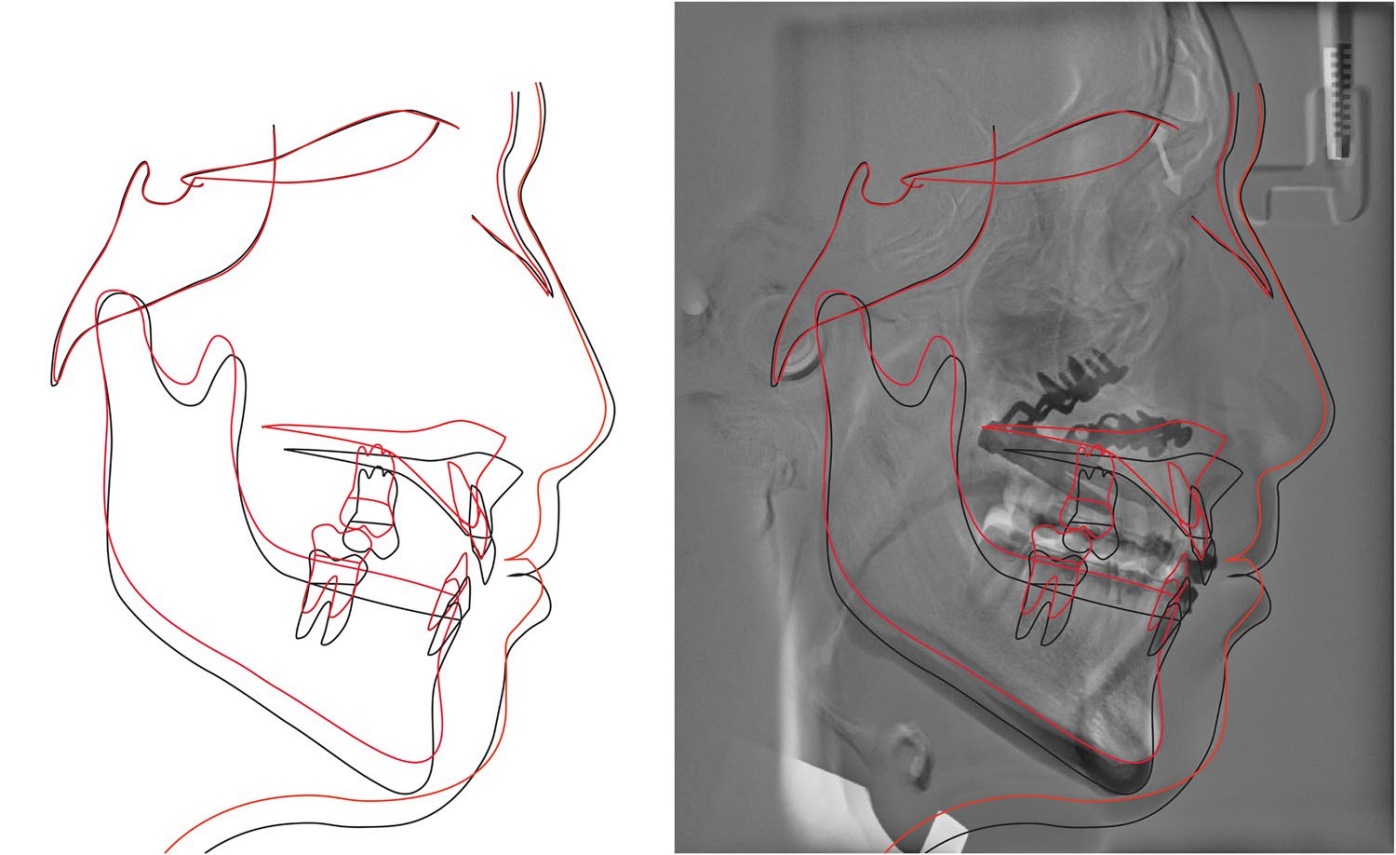
Illustration of relapse in the superimposed radiographs acquired at T1 and T2. The drawings of the cephalograms illustrate the superimposed skeletal situations at T1 (black lines) and T2 (red lines). The superimposed radiographs illustrate the same skeletal situation with a relapse between T1 and T2. The analyses are performed as described in Fig. 4. The TW plane method was used

Table 1 shows the cephalometric parameters used in this study. Figure 4 shows the cephalometric landmarks, refence lines and measures used. Six angular and linear parameters were used to describe the skeletal changes related to the two surgical treatments. As there was a short time between T0 and T1 during which no growth-related positional changes of the reference landmarks were expected to occur, conventional cephalometric measurements were performed [20]. However, at T2, a superimposition-based cephalometric method was used to assess the stability of the treatment [20]. Superimposition was performed on the anterior cranial base using the Tuberculum Sella-Wing (TW) plane method (Fig. 5) [20].

**Table 1:** Definitions of the cephalometric parameters used in this study

Cephalometric parameter	Definition
SNA (°)	Angle between the Sella, Nasion, and A-point, identifying the skeletal sagittal relation of the maxilla to the anterior cranial base
A-NSLP (mm)	Horizontal distance between the A-point and vertical reference line (NSLP), identifying the skeletal sagittal changes in the anterior part of the maxilla at the A-point in relation to a vertical reference line
A-NSL (mm)	Vertical distance between the A-point and horizontal reference line (NSL), identifying the skeletal vertical changes in the anterior part of the maxilla at the A-point in relation to the anterior cranial base
NL/NSL (°)	Angle between the maxillary plane and horizontal reference line (NSL), identifying the rotation of the maxilla in relation to the anterior cranial base
ANS-NSL (mm)	Vertical distance between the anterior nasal spin and horizontal reference line (NSL), identifying the skeletal vertical changes in the anterior part of the maxilla at the anterior nasal spin in relation to the anterior cranial base
PNS-NSL (mm)	Vertical distance between the posterior nasal spin and horizontal reference line (NSL), identifying the skeletal vertical changes in the posterior part of the maxilla at the posterior nasal spin in relation to the anterior cranial base

### Reliability

One of the authors (NA-T) performed all of the cephalometric analyses, both the conventional and superimposition-based techniques, using the digital analysis programme FACAD® (cephalometric software ver. 3.9.2.1133; Ilexis AB, Linköping, Sweden). The superimposition-based cephalometric measurements were repeated by the author (NA-T) 3 weeks later for 16 radiographs from eight randomly selected patients at T0 and T2, to assess intra-observer reliability. Another author (MR) performed tracings of the same 16 radiographs, to estimate inter-observer reliability.

### Statistical analysis

The data were statistically analysed using the IBM SPSS ver. 28 software. Paired sample *t*-tests were used to evaluate movement between T0 to T2 and T1 to T2. Mann–Whitney U-test was used to analyse differences in skeletal relapse rate between age groups. A P-value < 0.05 was considered statistically significant. The intra-class correlation coefficient (ICC) was used to evaluate the levels of intra- and inter-observer agreement of the superimposition-based cephalometric measurements.

## Results

The movements of the skeletal structures of the maxilla achieved through surgery were expressed in the sagittal and vertical planes using the recently described superimposition-based cephalometric analysis (Table 2).

**Table 2:** Summary of changes following maxillary distraction osteogenesis (DO) or Le Fort I osteotomy (CO). Values shown are means (standard deviation). Linear (mm) and angular (°) changes, and skeletal relapse are expressed in percentages. Statistically significant differences (in ***bolded italics***) are noted between changes in the position of the maxilla from T0 to T1, T0 to T2 and T1 to T2

	**Mean movement**	**Skeletal relapse**	**Mean movement**	**P-value**	**P-value**
**Parameters**	**T0 to T1**	**T1 to T2**	**T0 to T2**	**T0 to T2**	**T1 to T2**
**A sag / NSLP (mm)**					
**DO**	7.0 (2.3)	16%	5.9 (3.0)	** < *0.001***	***0.040***
**CO**	2.6 (2.6)	15%	2.2 (2.6)	***0.036***	*0.418*
**A ver / NSL (mm)**					
**DO**	4.3 (4.3)	36%	2.8 (3.0)	***0.009***	*0.059*
**CO**	2.9 (4.2)	32%	2.0 (3.9)	*0.164*	*0.122*
**ANS-NSL (mm)**					
**DO**	5.0 (4.0)	54%	2.3 (3.1)	***0.026***	***0.002***
**CO**	3.1 (5.0)	24%	2.4 (4.7)	*0.164*	*0.148*
**PNS-NSL (mm)**					
**DO**	1.7 (2.3)	+ 14%	1.9 (2.6)	***0.024***	*0.737*
**CO**	1.4 (2.7)	48%	0.7 (2.4)	*0.399*	*0.152*
**SNA (°)**					
**DO**	7.1 (2.4)	17%	5.9 (2.8)	** < *0.001***	***0.030***
**CO**	2.9 (2.1)	19%	2.3 (2.1)	***0.010***	*0.263*
**NL/NSL (°)**					
**DO**	3.8 (3.9)	87%	0.49 (3.4)	*0.623*	***0.002***
**CO**	1.7 (4.7)	+ 21%	2.0 (5.5)	*0.305*	*0.666*

The sagittal mean movements forward from T0 to T2 in patients treated with DO and CO were 5.9 mm and 2.2 mm, respectively, with a skeletal relapse rate of 16% in the DO group and 15% in the CO group. The sagittal movement between T0 and T2 was significant after both DO and CO (P < 0.001 and P = 0.036, respectively), whereas the sagittal skeletal relapse between T1 and T2 was significant for DO but not for CO (P = 0.040 and P = 0.418, respectively).

Maxillary movements in the vertical plane between T0 and T2 were 2.8 mm and 2.0 mm after DO and CO, respectively, with a skeletal relapse rate of 36% for DO and 32% for CO. The vertical movement from T0 to T2 was significant in DO (P = 0.009) but not in CO (P = 0.164). The vertical skeletal relapse between T1 and T2 was not significant after DO or CO (P = 0.059 and P = 0.122, respectively).

A subgroup analysis on age in relation to skeletal relapse rate showed a mean of 13% sagittal skeletal relapse rate in the younger group and 18% in the older group, which was not a significant difference (P = 0.257). No significant difference was found in vertical skeletal relapse rate either, the younger group showed 36% relapse and in the older group 33%, (P = 1.000).

Both the intra-observer and inter-observer reliability levels were considered excellent (ICC = 0.994 and ICC = 0.993, respectively), as defined by Koo et al., who consider ICC values of 0.6–0.8 to represent good agreement and ICC values of 0.8–1.0 to show excellent agreement [26].

## Discussion

The present study shows that sagittal advancement of the maxilla in patients with various CLP diagnoses can be achieved by either DO or CO with a high level of stability over time. Furthermore, sagittal movement is stable in cases treated with DO, although the advancement is extensive, and the age of the patient at the time of surgery do not affect the risk for skeletal relapse significantly.

The analyses of the surgical changes after the orthognathic treatment were performed using a new digital superimposition-based cephalometric method that uses stable landmarks for superimposition [20]. Previous studies have demonstrated that the reference landmarks sella and nasion used in conventional cephalometric analyses are displaced over time. For example, the nasion landmark can exhibit vertical and sagittal age-related displacements, such that the vertical and sagittal changes can be under-estimated or over-estimated [20, 27, 28].

Assessments of surgical corrections to 3D objects using 2D images has known limitations [29]. For example, we did not assess possible transversal changes. Low-dose cone beam computed tomography (CBCT) allows examinations of 3D changes in the craniofacial skeleton, e.g., after orthognathic treatment, through the extraction of 2D images without variations in magnification. However, low-dose CBCT examinations provide 15–26-fold higher radiation doses compared to lateral cephalograms [30]. Furthermore, when the results of cephalometric measurements performed on conventional lateral cephalograms were compared with re-constructed CBCT 2D images, no significant differences were found [31–33]. In a recent systematic review, it was concluded that cephalometric analyses using CBCT-scans have similar levels of accuracy to cephalometric analyses using conventional 2D cephalograms. CBCT was only recommended when the conditions required more-advanced diagnostics for treatment planning [34]. The higher radiation dose from CBCT explains why conventional cephalometry remains the preferred option in many countries, since justification and optimisation of radiographic examinations are required; these include keeping the radiation dosages as low as diagnostically acceptable, especially for young patients [35].

When analysing changes from T1 to T2 in the sagittal plane, the DO group showed significant skeletal relapse, whereas the CO group did not. This may be attributed to the fact that the patients in the DO group all had an anterior advancement of the maxilla, whereas three out of nine patients in the CO group were only treated with vertical movement of the maxilla (and no advancement in the sagittal plane). Therefore, three patients in the CO group showed no relapse in the sagittal plane, whereas all the patients in the DO group showed relapse to some extent.

Earlier studies have reported that skeletal relapse correlates with the extension of surgical advancement, with greater movement leading to a higher risk of skeletal relapse [10, 11]. This is, however, a controversial topic because other studies have concluded the opposite. Watts et al. [36] compared two groups with different extension of skeletal movements (mean movements of 4.9 mm and 9.8 mm) and could not find a correlation between skeletal relapse and the magnitude of the movement.

Due to the large differences in surgical movement observed between the different surgical procedures (DO and CO) in the present study, it is difficult to compare the relapse rates for the two techniques, although we conclude that relapse occurs with both techniques.

Chua et al. [14] have reported that CO results in a sagittal skeletal relapse rate of 31%, whereas DO only results in a relapse rate of 8% at 5 years post-surgery. A possible explanation for this is that the extension of surgical movement (in millimetres) was similar for the two groups (CO, 6.84 mm and DO, 7.04 mm). However, we can conclude that DO enables more-comprehensive surgical corrections in patients who have extensive skeletal discrepancies, without compromising post-operative skeletal stability.

In the present study, we detected more-extensive skeletal relapse in the vertical plane compared to the sagittal plane. This result highlights that surgical vertical movement of the maxilla downwards is less-stable than anterior advancement in patients with CLP. Our finding of skeletal stability after surgical movement of the maxilla confirms previously reported findings. Bailey et al. [9] have shown that the levels of stability differ according to the directions of the movements. On the one hand, surgical advancement of the maxilla is considered stable within a magnitude of anterior movement of < 8 mm [9]. On the other hand, surgical movement of the maxilla downwards in the vertical plane carries a high risk of skeletal relapse [9]. Other studies have drawn the same conclusion, whereby the rate of skeletal relapse in the sagittal plane was 17%–37% and the rate of skeletal relapse in the vertical plane was in the range of 26%–65% [24, 37–39].

A recent study conducted by Jang et al. [37] in which they used only CO and evaluated skeletal stability after surgery for 19 patients with CLP, the skeletal relapse rate was 18% in the sagittal plane after a mean forward advancement of 5.6 mm. The skeletal relapse rate after vertical movement was 26% following a mean downwards movement of 1.8 mm and SNA-angle relapse of 19%. That sagittal skeletal relapse rate is in line with our results, although the vertical relapse rate is lower. One explanation could be that the surgery-induced downwards movement in our study was greater (means of 4.3 mm for DO and 2.9 mm for CO) [37].

A systematic review [39] has reported higher rates of skeletal relapse for both the sagittal (37%) and vertical planes (65%) than those reported in our study. While the extensions of the sagittal and vertical movements were comparable to those seen in the present study, the follow-up times varied from 1 to 5 years. The mean follow-up time in the present study was 18 months, which is shorter than the follow-up times in of the most studies included in the review. It would be interesting to perform long-term follow-up studies of our patients, to evaluate whether the skeletal relationships are stable in the long-term.

In the present study, 8% of the patients born with clefts underwent orthognathic surgery due to skeletal discrepancies. In a study of 538 patients with CLP [6], 27% would benefit from orthognathic surgery based on their cephalometric analyses. In addition, Cohen et al. [7] have reported that while orthognathic surgery is recommended in 24%–26% of cases, only 11%–14% of patients are operated. De Luke et al. [5] have reported an orthognathic surgery rate of 25%. The reasons for the differences in the surgical rates between our study population and others should be examined further. One can speculate about the relevance of the surgical techniques used and the age of the patients at the time of the primary closure of the clefts.

In a literature review of DO and CO performed on patients with CLP, no clear evidence was found regarding which technique provides the optimal aesthetic outcome and functional improvement [40]. Those authors speculated that DO causes higher levels of anxiety and distress in patients, as compared to patients who undergo CO [40]. Chua et al. have also stated that DO may induce short-term distress in patients [41]. DO is an intensive treatment for the patient, with several care contacts during the active distraction. We found no difference in the relative relapse rate between DO and CO. However, it is difficult to draw firm conclusions from the results in the present study, since the magnitude of the mean anterior advancement differed between the groups. When making treatment decisions, the treatment load must be taken into consideration for each individual patient. It would be interesting to evaluate the experiences of patients with CLP after they have undergone different surgical procedures.

A systematic review published by Kloukos et al. [3] concluded that using a distraction device in conjunction with orthognathic surgery is not the gold standard for treatment. The review revealed a lack of high-quality randomised controlled studies evaluating orthognathic surgery compared to DO. Only one small randomised, controlled trial was included in the review. This highlights the need for more prospective research with larger cohorts, perhaps involving a multi-centre trial, with the aim of evaluating the differences between the post-operative outcomes of CO and DO [3].

### Strengths

The strengths of this study are that all surgical procedures and radiographical examinations were performed at the same cleft treatment centre and that all follow-ups were standardised.

### Limitations

Assessment of surgery using 2D imaging has known limitations. The number of patients included in this study was low, which makes it difficult to draw generalising conclusions. Since a new digital superimposition-based method was used for the analyses of surgical changes, comparisons with other studies need to be approached with caution.

## Conclusion

This superimposition-based cephalometric follow-up of two orthognathic surgical procedures in patients with CLP shows that sagittal advancement of the maxilla can be achieved with stable outcome; even extensive advancement can be attained, in this study with maxillary distraction. In contrast, vertical downward movement is unstable, independent of surgical procedure. Further randomised studies with larger study groups are warranted.

## Data Availability

No datasets were generated or analysed during the current study.
